# The influence of migrant children's identification with the college matriculation policy on their educational expectations

**DOI:** 10.3389/fpsyg.2022.963216

**Published:** 2022-08-23

**Authors:** Jingjing Xu, Cixian Lv

**Affiliations:** Institute of Educational Economics and Management, Normal College, Qingdao University, Qingdao, China

**Keywords:** college matriculation policy, policy identification, migrant children, educational expectation, influencing mechanism, moderating effect

## Abstract

Based on the theoretical framework of cultural reproduction theory and ecosystem theory, this paper explores the impact of migrant children's identification with the college entrance examination policy on their educational expectations and the associated underlying mechanisms from the micro, meso, and macro levels. In total, 1,770 questionnaires were collected from students, and 436 people were interviewed, including students, their teachers, and their parents. They are all from China. Through multidimensional analysis, the results indicated that both individual academic achievement and family social capital have positive impacts on migrant children's educational expectations and that social class segregation in school and perceived social discrimination have negative impacts on their educational expectations. Migrant children's identification with the policy has a significant positive impact on these children's educational expectations. Their identification with the policy enhances the positive impact of individual academic achievement and family social capital on their educational expectations and partially weakens the negative impact of social class segregation in school and perceived social discrimination on their educational expectations. The analysis suggests that college matriculation policy for migrant children drives a compensation mechanism that involves the “principle of justice”, a cultural mechanism that involves “promoting learning through examinations”, and an institutional mechanism involving “urban-rural integration” to increase educational expectations. This study enriches and develops the expectation theory of migrant children and provides a policy reference for local governments to improve their policies for college entrance examinations for migrant children and to promote household registration system reform.

## Introduction

According to the 2020 National Education Development Statistical Bulletin, among the students in the compulsory education stage, there were 14,297,300 children of migrant workers with about 9.1% (10.348 million in elementary school with about 9.6% and 3.948 million in junior high school with about 8%) (Ministry of Education of the People's Republic of China, 2020). As such, there are currently a large number of migrant children in cities. Whether migrant children can obtain a high-level education is an important social issue that needs to be considered in modern society because it directly affects the overall level of human resources in the country. Therefore, subject to the household registration system, how migrant children can take the entrance examination for higher education in the place of inflow is an important item in China (Feng and Liu, 2018). In this context, China has introduced the college matriculation policy. In addition, a large number of domestic and foreign studies have shown that human capital is the main determinant of modern social stratification and mobility and that educational expectations have a direct, stable, and effective explanatory power for the acquisition of human capital (Davis-Kean, 2005; Brand and Xie, 2010). Therefore, studies of the impact of the college matriculation policy for migrant children on their educational expectations have very important practical relevance.

In the environment surrounding migrant children, there are various factors that affect their educational expectations, such as individual academic achievement, family social capital, social class segregation in schools, and perceived social discrimination. Relevant studies from different countries have investigated how the educational expectations of migrant children are affected in such contexts (Goyette and Xie, 1999; Yan and Lin, 2005; Berry et al., 2006; Wu and Huang, 2017). However, existing studies have not yet clearly elucidated whether migrant children's identification with the college matriculation policy has had a significant impact on their educational expectations, nor have studies investigated this impact from the perspective of ecosystem theory.

This study will further explore this issue through a combination of quantitative and qualitative methods. Specifically, drawing on questionnaire data and students' essays about their educational experience, stratified sampling was used in this study to select students in grades 8–11 in 11 regions of 10 provinces. These provinces have substantial migrant population groups and were among the first to implement the college matriculation policy specifically for migrant children. And all 11 regions studied have a high concentration of migrant children. Subsequently, combining correlation analysis, regression analysis, and moderating effect tests, this study analyses the mechanism by which migrant children's identification with the college matriculation policy impacts their educational expectations. The results of this study can enrich both the theories related to the sociology of education in the context of college entrance examination reform as well as the theoretical framework and evaluation system of educational policy analysis. Furthermore, the results can contribute to revealing the mechanism by which migrant children's identification with the college matriculation policy influences their educational expectations.

## Theoretical foundation and research hypothesis

In the context of China's social transformation and large-scale population migration, the extent to which the social mobility of migrant children who are enrolled in urban schools or schools specifically for migrant workers is constrained, and whether there is a possibility of social mobility outside the logic of social class reproduction, remains unclear. The research about this should pay more attention to the cultural mechanisms and ecosystems behind the differences in educational outcomes. The “cultural reproduction theory” (Bourdieu, 1997) and the “ecosystem theory” (Bronfenbrenner, 1979), which originated in Western countries, have important theoretical guiding significance with regard to this issue. First, these theories shifted the focus of theoretical attention from the analysis of differences in educational outcomes to the mechanism of the educational process. Second, these theories provide a multidimensional perspective, from the micro to macro levels, for the analysis of influencing mechanisms. Finally, the theories provide a theoretical analysis framework revealing the mechanism by which educational policies impact the educational expectations of migrant children.

### Cultural reproduction theory

The cultural reproduction theory by Pierre Bourdieu, a French sociologist, originated in the 1970s. In contrast to the popular belief of the time, Bourdieu (1997) believed that school is the main arena which can produce and reproduce culture and social inequality (p. 192–200). Bourdieu has introduced three key concepts in his cultural reproduction theory. One concept is “cultural capital”. Lamont and Lareau (1998) believe that cultural capital is “widely accepted high-level cultural signals, such as attitudes, behaviors, preferences, formal knowledge, and diplomas. These signals are used to distinguish between cultural and social”. Another concept is “habitus”. Nash (1990) considered that habitus in Bourdieu's work refers to a system of embodied dispositions that generate practice in accordance with the structural principles of the social world. Therefore, habitus constitutes the principles guiding people's behaviors and tends to reproduce ambitions, feelings, and practices. Children of the advantaged class with specific cultural capital often have a certain habitus, which will reflect the values and behavior patterns recognized and appreciated by the school and the social environment, thereby helping them to establish higher educational expectations and obtain good educational achievement. The last concept is “field”. Actually, Bourdieu's work has provided a series of analyses of different social fields. For example, Bourdieu and Terdman (1987) analyzed what they term the “juridical field”. They believed that “a ‘field' is an area of structured, socially patterned activity or ‘practice', in this case disciplinarily and professionally defined.” And this also applies to the “school field”. In conclusion, Bourdieu and Wacquant (1992) believed that “practice” is the result of a person's “habitus” and his or her position in the field (capital), both of which operate together in a certain social environment (field).

The cultural reproduction theory has the following main viewpoints. First, the distribution of cultural capital is unequal. Children of the disadvantaged class can only obtain a small amount of cultural capital in a single form from family and residential communities; in contrast, children of the advantaged class can obtain rich and diverse forms of cultural capital from family and residential communities (Barn, 2010; Wu et al., 2014). Therefore, before children enter the formal education system, there has been a large degree of differentiation in cultural taste, rule identification, educational expectations and even behavioral habits (Li, 2013; Mu and Jia, 2016). Second, school education expands the difference in cultural capital. Bourdieu (1979) is persuaded that in contemporary societies social classes preserve a strong cultural identity (Schwartz, 1997). Bourdieu (1997) believes that school culture is the culture of the middle class or other ruling classes. Children who grow up in this cultural background are in a favorable position in school-based education, while children of the working class and other lower classes often do not adapt to this culture, becoming laggards in school-based education. In addition, children with a larger amount and form of cultural capital are more likely to understand the content of the courses taught at a school, more easily interact with peers, and know how to better use school resources to fight for their own interests. These groups with higher cultural capital can often develop higher educational expectations through school-based education, potentially translating into higher academic achievements. Third, cultural reproduction achieves hierarchical reproduction. DiMaggio (1982) made a hypothesis in his article, “Returns to cultural capital are highest for students from high-status families and least for students from low-status families”. Bourdieu (1997) proposes that schools with the cultural characteristics of the ruling class and a differentiated education system have become the cultural capital that continues to widen the cultural differences among different classes. This cultural capital plays the role of “gatekeeper” in maintaining class boundaries and completing the process from cultural reproduction to class reproduction by transforming academic advantages into professional advantages.

### Ecosystem theory

The ecosystem theory proposes that individual development is always nested in a series of mutual influences. That is, the interaction between the individual and the environmental system and the interaction between environmental systems together affect the development of individual cognition and behavior (Bronfenbrenner, 1979). According to this theory, there are microsystems, outer-layer systems, macrosystems, and a chronosystem. Migrant children are also in an educational ecosystem like other children, and their educational expectations are also affected by changes in various subsystems caused by changes in the chronosystem. Therefore, we borrowed the ecosystem theory to construct an ecosystem of educational expectations mainly from the micro level (individuals), the meso level (families and schools), and the macro level (social policies).

#### Individual academic achievement at the micro level

An individual's academic achievement is the logical starting point for educational expectations. According to the “looking-glass self” theory of social psychology, an individual's self-evaluation is based on the judgments they receive from others as a “mirror”, and the individual understands and grasps himself or herself through this “mirror” (Cooley, 2015). Goyette and Xie (1999) analyzed the educational expectations of Asian American students in high schools and found that students with better academic achievement were more positively strengthened in their interactions with teachers, parents, and other peers, thereby promoting higher levels of educational expectations. However, students with poor grades often receive negative feedback in the process of interacting with others; therefore, they lose confidence and generally hold low educational expectations. Rutchick et al. (2009) propose that there is a significant independent influence between students' academic achievement and educational expectation. According to the cultural adaptation theory, as migrant children of disadvantaged groups, although there is heterogeneity and discontinuity between their family cultural background or early cultural acquisition and the urban school culture, there is still a certain correlation between their academic achievements and educational expectations (Segall et al., 1998). Based on the studies above, the following hypothesis is proposed:

H1. The existing academic achievements of migrant children have a significant positive impact on their educational expectations.

#### Family social capital at the micro level

Since the economic reforms, rural people have been permitted to move to urban areas. The immigrant family mentioned in this paper refer to this category who do not have rich family capital; therefore, can their family social capital effectively predict their educational expectations? James (2001) proposes that the value standards of migrant families for education often reflect the principles of “safety first” and “risk aversion” (p. 33). However, more scholars believe that although minority groups and migrant workers lack family social capital, they still maintain the confidence to achieve social mobility through education. Yan and Lin (2005) argue that family social capital, such as participation in education by people in lower classes, is very beneficial for children to establish good education expectations. Gonzalez-Dehass et al. (2005) propose that the participation behaviors of self-supporting parents can significantly increase children's educational expectations, with further improvements in reading, communication, and participation in sports activities. Xu et al. (2017) found that parental rearing styles and emotional attitudes can reduce the level of social anxiety in migrant children but that excessive protection will increase the level of social anxiety in children. The family has a very important impact on migrant children. In the interviews in this study, we found that many parents of migrant children believed that although it is difficult for the family to provide all types of capital needed for their children's educational expectations, if there are no expectations, the children will not be motivated to learn, and life will become more difficult in the future. The family social capital of migrant children affects the transmission of intergenerational values and educational preferences. Based on the above analysis, the second hypothesis is proposed:

H2. Family social capital has a significant positive impact on the educational expectations of migrant children.

#### Social class segregation in schools at the meso level

Currently, the schools that migrant children attend are often marginalized public schools or low-class schools for children of migrant workers (Chen and Feng, 2013). The Coleman report noted that there was a correlation between educational segregation and educational consequences and that educational integration had different effects on the academic achievements of students of different races and had a positive effect on the academic achievements of black students but did not have significant effects on the academic achievements of white students (Coleman et al., 1966). Therefore, it can be inferred that different degrees of household registration integration in China affect migrant children, with no significant negative impact on non-migrant students. Wu and Huang (2017) used the class composition at school as the core independent variable to explore the impact of class segmentation in schools on students' educational expectations. Model analysis revealed that student class heterogeneity within schools was a significant factor, i.e., for every 1-unit increase in the standard deviation of the within-group composite socioeconomic status index, the odds of students expecting to attend college increased by 7.3%. Additionally, there were group differences in the effect of class heterogeneity on educational expectations, with a 1-point increase in the mean class status index associated with a 0.018-year increase in educational expectations for students at the bottom of the achievement scale. In summary, an increase in the average class level and class heterogeneity in schools and classes will affect the educational expectations of the students, especially for those students with low grades or low cognitive ability. The gap between such students and students with high educational expectation scores needs to be decreased significantly. Based on the above analysis, the following hypothesis is developed:

H3. Social class segregation in schools has a significant negative impact on the educational expectations of migrant children.

#### Perceived social discrimination at the macro level

Domestic and international studies on the impact of perceived discrimination on the children of immigrants have focused on psychological status, cultural adaptation or social integration and have reached a relatively consistent conclusion that the impact of perceived discrimination is generally negative. Lazarus and Folkman (1984) argue that discrimination is a source of stress for individuals and that individuals need to have certain resources to cope with it. However, due to the lack of an adequate social support system for migrant children, they are prone to a series of negative reactions under this source of stress, for example, anxiety and depression. Xiong et al. (2021) proved that there is a significant positive correlation between perceived social support and the pro-social tendencies of migrant children. Berry et al. (2006) studied 5,366 immigrant adolescents from 13 countries and found that perceived discrimination was significantly negatively correlated with their sociocultural adaptation status. Berkel et al. (2010) found that a discriminatory experience had a partial impact on mental health and the experience caused changes in academic self-efficacy and academic performance. The ecosystem theory proposes that the development of individuals is nested in a series of interacting environmental systems. That is, the interaction between the individual and the environmental system and the interaction between environmental systems together affect the development of individual cognition and behavior (Bronfenbrenner, 1979). The psychological state, school adaptation, and social integration of migrant children under the influence of perceived discrimination will inevitably affect their educational expectations. Based on the above analysis, the fourth hypothesis is proposed:

H4. Perceived social discrimination has a significant negative impact on the educational expectations of migrant children.

### Correlations between migrant children's identification with the college matriculation policy and their educational expectations

Will children's identification with the college matriculation policy have an impact on their educational expectations? Many studies have shown that there is a significant negative correlation between segregation, such as regional segregation, racial segregation and educational segregation, and students' educational expectations (Alan, 1959; Tienda, 1998). Scholars in China have also found that there is a correlation between migrant children's identification with the college matriculation policy and their educational expectations. Due to the special household registration system in China, before the implementation of the college matriculation policy for migrant children, migrant children in schools were marginalized and treated differently (Guo, 2007). This is because household registration (*hukou* system) results in different cultural capital and determines eligibility and access to government provision of social services and benefits (Cheng and Selden, 1994). There is a big huge gap between urban household registration and agricultural household registration. This embarrassing situation has greatly weakened the enthusiasm for learning by migrant children and hindered the possibility of them establishing good educational expectations. Wu (2011) found that there was a significant education gap among people with different household registrations and that migrant children who could not take local entrance examinations were disadvantaged in terms of expected years of education. Song et al. (2017) conducted a long-term follow-up survey of migrant children and found that only 56.12% of the children in the survey had expectations of attending college or higher levels of education. He proposed that the college entrance examination policy, which was based on household registration, was the institutional factor causing low educational expectations. The boundary permeability theory proposes that only a flexible social structure that is dynamic and permeable can prompt a disadvantaged group to obtain a new dimension of intergroup comparison through individual efforts to change an individual's survival state (Tajfel, 1978). Before the implementation of the college matriculation policy for migrant children, migrant children encountered dual obstacles, i.e., the household registration system and the education policy, and the impermeability of the group boundary seriously dampened their psychological capital; therefore, it was impossible for them to form positive educational expectations. The introduction of the policy, although the conditions of policy vary from place to place, undoubtedly enhances the permeability of intergroup boundaries and provides migrant children with an opportunity to establish positive educational expectations. Aligned with the analysis above, this paper introduces migrant children's identification with the college matriculation policy as a variable and develops the following hypotheses:

H5. Policy identification has a significant positive impact on the educational expectations of migrant children.

H6a. Policy identification has a moderating effect between the individual academic achievement and educational expectations of migrant children.

H6b. Policy identification has a moderating effect between the family social capital and educational expectations of migrant children.

H6c. Policy identification has a moderating effect between social class segregation in schools and educational expectations of migrant children.

H6d. Policy identification has a moderating effect between the perceived social discrimination and educational expectations of migrant children.

## Materials and methods

### Research background

In this study, we used stratified sampling to investigate 8th to 11th grade students, as these students will take the entrance examination in the near future. The samples were collected from 15 schools in 11 regions of 10 provinces that have substantial migrant population groups and were among the first to implement the college matriculation policy specifically for migrant children. All 11 regions have a high concentration of migrant children. Among the schools surveyed above, 11 were public schools, and four were private schools. The research team conducted on-site application of the questionnaires and then used list-wise deletion to further screen the sample.

A total of 1,770 valid questionnaires were obtained, all of which were valid. As shown in Table 1, more than half of the respondents were male (54.1%), and 75.1% (*n* = 1,329) of the sample attended local public school. There are two types of household mobility, and 79.4% (*n* = 1,405) of the sample had undergone rural to urban mobility. With regard to personnel structure of residence, 38.6% (*n* = 683) of the respondents reported that the numbers of locals and outsiders were “about the same”. Nearly half of the sample had lived in their current region for 3–4 years (*n* = 864, 48.8%).

**Table 1:** Demographic characteristics of the sample.

**Variable**	***n***	**Percentage**
**Sex**		
Female	813	45.9%
Male	957	54.1%
**Type of household mobility**		
“Rural-urban” mobility	1,405	79.4%
“Town-town” mobility	365	20.6%
**School type**		
Voluntary school (migrant children)	441	24.9%
Local public school	1,329	75.1%
**Personnel structure of residence**		
Mostly locals	481	27.2%
About the same	683	38.6%
Mostly outsiders	606	34.2%
**Duration of residence**		
1–2 years	201	11.4%
3–4 years	864	48.8%
5–6 years	500	28.2%
≥7 years	205	11.6%

N = 1,770.

On the basis of factors such as household registration type, family background, and school type, the research team randomly selected migrant children, parents, and teachers for in-depth interviews. Altogether, the research team conducted interviews with 312 migrant children, 89 parents of migrant children, and 35 teachers.

In addition, the data were supplemented using the qualitative research tool “proposition composition”, which required the students to write about their “learning experience and future expectations in a place far from home”. The students' essays responded to the following questions: (1) What differences do you see between yourself and other students with regard to school and life? (2) Will you take the college entrance examination in the city where you live? What are the reasons for your choice? (3) What education level do you want to achieve? What type of work do you want to do in the future? What social achievements do you want to achieve? Overall, a total of 293 propositional essays were collected.

This study is mainly quantitative and is complemented by qualitative research methods. On the one hand, the quantitative method based on surveys was adopted to determine whether and the extent to which the college matriculation policy for migrant children has had an impact on their educational expectations. On the other hand, to explore how the matriculation policy affects children's educational expectations, qualitative methods based on in-depth interviews and proposition compositions are used to explore the mechanism underlying this impact. And then, we summarize three mechanisms which are expressed in Section Discussion.

### Instrumentation

This study included a total of six variables: (a) migrant children's identification with the college matriculation policy (ICMP); (b) migrant children's educational expectations (EE); (c) individual academic achievement (IAA); (d) family social capital (FSC); (e) social class segregation in schools (SCS); and (f) perceived social discrimination (PSD). These variables were developed based on prior literature and the interviews conducted in this study. The items were rated on a 5-point scale ranging from 1 (strongly disagree) to 5 (strongly agree).

#### Migrant children's identification with the college matriculation policy

Migrant children's identification with the college matriculation policy refers to their psychological acceptance and identification with the policy. This sentiment is expressed both in terms of belief in the spirit and value of the college matriculation policy for migrant children and in terms of loyalty and support for this policy. Based on a literature review (Wu and Zhu, 2016), we compiled a questionnaire on policy identification. The Cronbach alpha value was 0.965, and the corrected item-total correlation (CITC) value for each measurement item was >0.5. A validity test was conducted through confirmatory factor analysis, and the combined reliability was 0.967, the construct validity was 0.768, and the first-order confirmatory factor model was consistent with the formal sample data.

#### Educational expectations of migrant children

The educational expectations of migrant children refer to their subjective cognition formulated on the basis of the objective reality and their life experience, that is, their belief and desire for some education results (including academic achievement, moral performance, interpersonal relationship, and social achievement) that may be realized after their efforts (Rodman and Voydanoff, 1978; Spenner and Featherman, 1978). Based on a literature review (Liu, 2013) and the actual situations of migrant children, we developed an educational expectation questionnaire with a Cronbach alpha value of 0.968. A confirmatory factor model of educational expectations was constructed. The combined reliability values for its four dimensions were 0.933, 0.873, 0.912, and 0.879, and the construct validity was 0.779, 0.635, 0.723, and 0.647, respectively. The second-order four-factor confirmatory factor model was consistent with the formal sample data.

#### Individual academic achievement

Individual academic achievement refers not only to achievement in major courses, such as Chinese, mathematics, and English, but also to learning ability, learning habits, learning motivation, and extracurricular learning. Based on a literature review (Cheung and Pomerantz, 2011), we developed an individual academic achievement scale; the Cronbach alpha value was 0.940, and the CITC value for each measurement item was >0.5. A confirmatory factor model of individual academic achievement was constructed, and its combined reliability was 0.940. The construct validity was 0.692, and the first-order confirmatory factor model was consistent with the formal sample data.

#### Family social capital

Family social capital reflects the various resources acquired or mobilized by family members in purposeful activities and embedded in the family social network and includes intra-family social capital, such as family economic capital and family cultural capital, as well as extra-family social capital, such as parental participation in education, the relationship between teachers and parents and the relationship between the student and his or her classmates. Based on literature review (Shi and Shen, 2007) and the actual situation for migrant children, we developed a family social capital scale, with a Cronbach alpha value of 0.929, and constructed a confirmatory factor model of family social capital, which had a combined reliability of 0.931 and a construct validity of 0.66. The first-order confirmatory factor model was consistent with the formal sample data.

#### Social class segregation in schools

Social class segregation in schools refers to the aggregation of students from different races and classes into different schools, resulting in insufficient uniformity in the distribution of student types among schools and significant class differences. Based on a literature review (Massey and Denton, 1988; Wu and Huang, 2017) and the actual situation for migrant children, we developed a scale for social class segregation in schools, which had a Cronbach alpha value of 0.852, and constructed a confirmatory factor model for class segregation in schools, which had a combined reliability of 0.881 and a construct validity of 0.599. The first-order confirmatory factor model was consistent with the formal sample data.

#### Perceived social discrimination

Perceived discrimination is a subjective experience relative to objective discrimination. It usually refers to an individual experiencing feeling of unfair or hurtful treatment due to his or her group (e.g., race, gender, or household registration status). This unfair treatment can manifest as a refusal attitude, behavior, or unreasonable system or policy in society. Based on literature review (Krahé et al., 2011) and the actual situation for migrant children, we developed a scale for perceived social discrimination. The Cronbach alpha value was 0.961. A confirmatory factor model was constructed for perceived social discrimination, with a combined reliability of 0.961 and a construct validity of 0.677. The first-order confirmatory factor model was consistent with the formal sample data.

### Data analysis

Using correlation analysis, we preliminarily verified the correlations between the independent variables above, including IAA, FSC, SCS, and PSD, and the different dimensions of the educational expectations of migrant children. Then, we performed regression models to further examine the moderating effect of migrant children's identification with the college matriculation policy on the relationships between all independent variables and different dimensions of educational expectations of migrant children. Because the educational expectations of migrant children include different degrees of differences in demography, these demographic variables were used as control variables in the regression model.

In addition, migrant children are not only the objects or subjects of education policy implementation but are also conscious social actors. Therefore, for the data obtained through in-depth interviews and the propositional compositions, qualitative research methods were used to further explain the reasons and mechanisms underlying the impact of their identification with the college matriculation policy on their educational expectations.

In summary, this study uses these three methods to analyse the impact of migrant children's identification with the college matriculation policy on their educational expectations and the associated underlying mechanism.

## Results

### Correlation analysis

To examine the linear relationship between the observed continuous variables, Pearson correlations were conducted. Table 2 provides specific correlations coefficient among the observed variables. As shown, most of the correlations held in the expected directions. ICMP, FSC, and IAA were positively related to the educational expectations of migrant children. In particular, FSC was the most positively related to educational expectations (AAE: *r* = 0.75, *p* < 0.01; MPE: *r* = 0.75, *p* < 0.01; IE: *r* = 0.63, *p* < 0.01; SAE: *r* = 0.67, *p* < 0.01). However, SCS and PSD were negatively related to the educational expectations of migrant children. Specifically, PSD was the most negatively related to educational expectations (AAE: *r* = −0.73, *p* < 0.01; MPE: *r* = −0.76, *p* < 0.01; IE: *r* = −0.76, *p* < 0.01; SAE: *r* = −0.75, *p* < 0.01).

**Table 2:** Correlation analysis of values.

	**(1)**	**(2)**	**(3)**	**(4)**	**(5)**	**(6)**	**(7)**	**(8)**	**(9)**
(1) ICMP	1								
(2) IAA	0.37^**^	1							
(3) FSC	0.37^**^	–	1						
(4) SCS	−0.59^**^	–	–	1					
(5) PSD	−0.71^**^	–	–	–	1				
(6) AAE	0.74^**^	0.74^**^	0.75^**^	−0.73^**^	−0.73^**^	1			
(7) MPE	0.68^**^	0.73^**^	0.75^**^	−0.73^**^	−0.76^**^	0.72^**^	1		
(8) IE	0.63^**^	0.76^**^	0.63^**^	−0.76^**^	−0.76^**^	0.89^**^	0.83^**^	1	
(9) SAE	0.67^**^	0.71^**^	0.67^**^	−0.73^**^	−0.75^**^	0.80^**^	0.82^**^	0.80^**^	1

N = 1,770.

AAE, academic achievement expectations; MPE, moral performance expectations; IE, interpersonal expectations; SAE, social achievement expectations.

** p < 0.01.

### Regression analysis

#### The impact of migrant children's identification with the college matriculation policy on their educational expectations at the individual level

##### Regression analysis of an individual academic achievement and the educational expectations of migrant children

As seen in Tables 3, 4, individual academic performance had the most positive predictive effect on IE (β = 0.87, *p* < 0.01) and also had a significant positive predictive effect on AAE (β = 0.70, *p* < 0.01), MPE (β = 0.69, *p* < 0.01), and SAE (β = 0.65, *p* < 0.01). H1 is supported by the data.

**Table 3:** Regressions results of the effect of demographic and related variables, IAA, FSC, SCS, and PSD on AAE and MPE.

	**AAE**				**MPE**			
	***β* (SD)**				***β* (SD)**			
**Variables**	**1**	**2**	**3**	**4**	**1**	**2**	**3**	**4**
(Constant)	1.17^**^ (0.38)	1.08^**^ (0.38)	5.21^**^ (0.30)	2.01^**^ (0.27)	1.70^**^ (0.40)	1.24^**^ (0.40)	5.79^**^ (0.31)	2.06^**^ (0.27)
Sex	0.00 (0.08)	0.01 (0.08)	0.03 (0.08)	0.03 (0.07)	−0.01 (0.08)	0.03 (0.08)	−0.01 (0.08)	−0.04 (0.08)
Type of household mobility	−0.01 (0.09)	−0.03 (0.09)	0.09 (0.09)	0.06 (0.08)	−0.11 (0.09)	−0.19 (0.09)	−0.01 (0.10)	−0.08 (0.09)
Personnel structure	0.23^**^ (0.05)	0.23^**^ (0.05)	0.23^**^ (0.06)	0.16^*^ (0.05)	0.24^**^ (0.06)	0.16^**^ (0.06)	0.24^**^ (0.06)	0.16^**^ (0.05)
Duration of residence	−0.19^**^ (0.06)	−0.17^**^ (0.06)	−0.25^**^ (0.06)	−0.16^*^ (0.06)	−0.25^**^ (0.06)	−0.21^**^ (0.06)	−0.30^**^ (0.07)	−0.20^**^ (0.06)
IAA	0.70^**^ (0.06)				0.69^**^ (0.06)			
FSC		0.72^**^ (0.06)				0.68^**^ (0.06)		
SCS			−0.79^**^ (0.08)				−0.78^**^ (0.08)	
PSD				−0.59^**^ (0.06)				−0.59^**^ (0.06)
**Δ*R*^2^**	0.60	0.60	0.56	0.59	0.59	0.61	0.57	0.66
**F**	165.34^**^	167.99^**^	155.03^**^	248.14^**^	166.35^**^	169.56^**^	157.32^**^	256.67^**^

Standard deviations in the brackets; 1 = a regression model including independent variable IAA and other control variables; 2 = a regression model including independent variable FSC and other control variables; 3 = a regression model including independent variable SCS and other control variables; 4 = a regression model including independent variable PSD and other control variables.

* p < 0.05,

** p < 0.01.

**Table 4:** Regressions results of the effect of demographic and related variables, IAA, FSC, SCS, and PSD on IE and SAE.

	**IE**				**SAE**			
	***β* (SD)**				***β* (SD)**			
**Variables**	**1**	**2**	**3**	**4**	**1**	**2**	**3**	**4**
(Constant)	1.05^*^ (0.44)	0.89^*^ (0.44)	6.25^**^ (0.35)	2.89^**^ (0.30)	1.77^**^ (0.40)	1.64^**^ (0.40)	5.70^**^ (0.31)	2.78^**^ (0.27)
Sex	0.04 (0.09)	0.03 (0.09)	0.04 (0.09)	−0.02 (0.83)	−0.02 (0.08)	−0.03 (0.08)	−0.01 (0.08)	−0.07 (0.08)
Type of household mobility	−0.08 (0.10)	−0.10 (0.10)	0.04 (0.11)	−0.03 (0.10)	−0.10 (0.10)	−0.11 (0.09)	−0.01 (0.09)	−0.04 (0.09)
Personnel structure	0.30^**^ (0.06)	0.30^**^ (0.06)	0.29^**^ (0.06)	0.13^*^ (0.06)	0.21^**^ (0.06)	0.21^**^ (0.06)	0.20^**^ (0.06)	0.12^**^ (0.05)
Duration of residence	−0.28^**^ (0.07)	−0.24^**^ (0.07)	−0.33^**^ (0.07)	−0.23^**^ (0.07)	−0.24^**^ (0.06)	−0.21^**^ (0.06)	−0.26^**^ (0.06)	−0.21^**^ (0.06)
IAA	0.87^**^ (0.07)				0.65^**^ (0.06)			
FSC		0.91^**^ (0.07)				0.68^**^ (0.06)		
SCS			−0.76^**^ (0.09)				−0.80^**^ (0.08)	
PSD				−0.59^**^ (0.07)				−0.59^**^ (0.06)
**Δ*R*^2^**	0.63	0.65	0.60	0.64	0.56	0.57	0.55	0.63
**F**	178.71^**^	183.98^**^	168.98^**^	261.23^**^	157.04^**^	160.28^**^	155.38^**^	220.12^**^

Standard deviations in the brackets; 1 = a regression model including independent variable IAA and other control variables; 2 = a regression model including independent variable FSC and other control variables; 3 = a regression model including independent variable SCS and other control variables; 4 = a regression model including independent variable PSD and other control variables.

* p < 0.05,

** p < 0.01.

##### Moderating effect of children's identification with the college matriculation policy on the relationship between individual academic achievement and educational expectations

As seen in Table 5, the positive predictive effect of ICMP on IE did not pass the significance test (β = 0.10, *p* > 0.05), while the product term of IAA and ICMP had a significant positive predictive effect on IE (β = 0.17, *p* < 0.01). Other dimensions all passed the significance test. First, ICMP had a significant positive predictive effect on AAE (β = 0.25, *p* < 0.01), meanwhile, the product term of IAA and ICMP also had a significant positive predictive effect on AAE (β = 0.13, *p* < 0.01). Second, ICMP had a significant positive predictive effect on MPE (β = 0.19, *p* < 0.01), and the product term of IAA and ICMP also had a significant positive effect on MPE (β = 0.15, *p* < 0.01). Finally, ICMP had a significant positive effect on SAE (β = 0.15, *p* < 0.05), and the product term of IAA and ICMP also had a significant positive effect on SAE (β = 0.13, *p* < 0.05). Therefore, most dimensions of H6a are supported by the data.

**Table 5:** Moderating effect of ICMP on the relationship between IAA and educational expectations.

	**AAE**		**MPE**		**IE**		**SAE**	
	***β* (SD)**		***β* (SD)**		***β* (SD)**		***β* (SD)**	
**Variables**	**1**	**2**	**1**	**2**	**1**	**2**	**1**	**2**
(Constant)	0.71 (0.38)	0.68 (0.38)	1.35^**^ (0.41)	1.32^**^ (0.40)	0.86 (0.46)	0.83 (0.45)	1.49^**^ (0.41)	1.46^**^ (0.41)
Sex	0.07 (0.08)	0.07 (0.08)	0.04 (0.08)	0.04 (0.08)	0.06 (0.09)	0.06 (0.09)	0.02 (0.08)	0.02 (0.08)
Type of household mobility	−0.09^**^ (0.09)	−0.07 (0.09)	−0.17 (0.09)	−0.15 (0.09)	−0.11 (0.11)	−0.09 (0.11)	−0.14 (0.10)	−0.13 (0.10)
Personnel structure	0.12 (0.06)	0.11 (−0.56)	0.16^**^ (0.06)	0.15^*^ (0.06)	0.25^**^ (0.07)	0.24^**^ (0.07)	0.15^*^ (0.06)	0.14^*^ (0.06)
Duration of residence	−0.18^**^ (0.06)	−0.20^**^ (0.06)	−0.24^**^ (0.06)	−0.26^**^ (0.06)	−0.28^**^ (0.07)	−0.29^**^ (0.07)	−0.24^**^ (0.06)	−0.25^**^ (0.06)
IAA	0.65^**^ (0.06)	0.65^**^ (0.06)	0.65^**^ (0.06)	0.65^**^ (0.06)	0.85^**^ (0.07)	0.85^**^ (0.07)	0.62^**^ (0.06)	0.62^**^ (0.06)
ICMP	0.25^**^ (0.05)	0.25^**^ (0.05)	0.19^**^ (0.06)	0.20^**^ (0.06)	0.10 (0.06)	0.11 (0.06)	0.15^*^ (0.06)	0.16^**^ (0.06)
IAA*ICMP		0.13^**^ (0.05)		0.15^**^ (0.05)		0.17^**^ (0.06)		0.13^*^ (0.06)
**Δ*R*^2^**	0.62	0.63	0.61	0.62	0.64	0.64	0.58	0.58
**F**	163.52^**^	157.73^**^	160.60^**^	155.20^**^	168.22^**^	162.35^**^	150.93^**^	145.97^**^

Standard deviations in the brackets; 1 = a regression model including independent variables IAA and ICMP; 2 = a model adds the product term of IAA and ICMP on the basis of model 1.

* p < 0.05,

** p < 0.01.

#### The impact of migrant children's identification with the college matriculation policy on their educational expectations at the family level

##### Regression analysis of family social capital and the educational expectations of migrant children

As seen in Tables 3, 4, household social capital had the most positive predictive effect on IE (β = 0.91, *p* < 0.01) and also had a significant positive predictive effect on MPE (β = 0.68, *p* < 0.01), AAE (β = 0.72, *p* < 0.01) and SAE (β = 0.68, *p* < 0.01). H2 is supported by the data.

##### Moderating effect of migrant children's identification with the college matriculation policy on the relationship between family social capital and educational expectations

As seen in Table 6, the positive predictive effect of ICMP on IE did not pass the significance test (β = 0.10, *p* > 0.05), while the product term of FSC and ICMP had a significant positive predictive effect on IE (β = 0.18, *p* < 0.05). Other dimensions all passed the significance test. First, ICMP had a significant positive predictive effect on AAE (β = 0.24, *p* < 0.01), meanwhile, the product term of FSC and ICMP also had a significant positive predictive effect on AAE (β = 0.14, *p* < 0.01). Second, ICMP had a significant positive predictive effect on MPE (β = 0.18, *p* < 0.01), and the product term of FSC and ICMP also had a significant positive effect on MPE (β = 0.15, *p* < 0.01). Finally, ICMP had a significant positive effect on SAE (β = 0.14, *p* < 0.05), and the product term of IAA and ICMP also had a significant positive effect on SAE (β = 0.14, *p* < 0.01). Most dimensions of H6b are supported by the data.

**Table 6:** Moderating effect of ICMP on the relationship between FSC and educational expectations.

	**AAE**		**MPE**		**IE**		**SAE**	
	***β* (SD)**		***β* (SD)**		***β* (SD)**		***β* (SD)**	
**Variables**	**1**	**2**	**1**	**2**	**1**	**2**	**1**	**2**
(Constant)	0.62 (0.38)	0.60 (0.37)	1.24^**^ (0.40)	1.21^**^ (0.40)	0.71 (0.45)	0.68 (0.44)	1.37^**^ (0.41)	1.34^**^ (0.40)
Sex	0.06 (0.08)	0.05 (0.07)	0.03 (0.08)	0.03 (0.08)	0.05 (0.09)	0.04 (0.09)	0.01 (0.08)	0.01 (0.08)
Type of household mobility	−0.10 (0.09)	−0.08 (0.09)	−0.19^*^ (0.09)	−0.16 (0.09)	−0.13 (0.10)	−0.11 (0.10)	−0.16 (0.09)	−0.14 (0.09)
Personnel structure	0.13^*^ (0.06)	0.12^*^ (0.05)	0.16^**^ (0.06)	0.15^*^ (0.06)	0.26^**^ (0.07)	0.25^**^ (0.07)	0.15^*^ (0.06)	0.14^*^ (0.06)
Duration of residence	−0.16^**^ (0.06)	−0.17^**^ (0.06)	−0.21^**^ (0.06)	−0.23^**^ (0.06)	−0.24^**^ (0.07)	−0.25 (0.07)	−0.21^**^ (0.06)	−0.22^**^ (0.06)
FSC	0.67^**^ (0.06)	0.67^**^ (0.06)	0.68^**^ (0.06)	0.68^**^ (0.06)	0.89^**^ (0.07)	0.88^**^ (0.07)	0.65^**^ (0.06)	0.65^**^ (0.06)
ICMP	0.24^**^ (0.05)	0.25^**^ (0.05)	0.18^**^ (0.06)	0.19^**^ (0.06)	0.10 (0.06)	0.11 (0.06)	0.14^*^ (0.06)	0.15^**^ (0.06)
FSC*ICMP		0.14^**^ (0.05)		0.15^**^ (0.05)		0.18^*^ (0.06)		0.14^**^ (0.05)
**Δ*R*^2^**	0.63	0.64	0.63	0.64	0.65	0.66	0.58	0.59
**F**	165.98^**^	160.14^**^	163.41^**^	157.98^**^	172.70^**^	166.61^**^	122.28^**^	148.88^**^

Standard deviations in the brackets; 1 = a regression model including independent variables FSC and ICMP; 2 = a model adds the product term of FSC and ICMP on the basis of model 1.

* p < 0.05,

** p < 0.01.

#### The impact of migrant children's identification with the college matriculation policy on their educational expectations at the school level

##### Regression analysis of class segregation in schools and the educational expectations of migrant children

As seen in Tables 3, 4, class segregation in schools had the most negative predictive effect on SAE (β = −0.80, *p* < 0.01) and also had a significant negative predictive effect on AAE (β = −0.79, *p* < 0.01), MPE (β = −0.78, *P* < 0.01), and SAE (β = −0.80, *p* < 0.01). H3 is supported by the data.

##### Moderating effect of migrant children's identification with the college matriculation policy on the relationship between class segregation in schools and educational expectations

As seen in Table 7, ICMP had a significant positive predictive effect on MPE (β = 0.82, *p* < 0.01), but the positive predictive effect of the product term of SCS and ICMP on MPE did not pass the significance test (β = 0.07, *p* > 0.05). ICMP had a significant positive predictive effect on SAE (β = 0.72, *p* < 0.01), but the positive predictive effect of the product term of SCS and ICMP on SAE did not pass the significance test (β = 0.07, *p* > 0.05). Other dimensions all passed the significance test. ICMP had a significant positive effect on AAE (β = 0.80, *p* < 0.01), and the product term of SCS and ICMP also had a significant positive effect on AAE (β = 0.15, *p* < 0.01). ICMP had a significant positive effect on IE (β = 0.85, *p* < 0.01), and the product term of SCS and ICMP also had a significant positive effect on IE (β = 0.22, *p* < 0.01). Half of the dimensions of H6c are supported by the data.

**Table 7:** Moderating effect of ICMP on the relationship between SCS and educational expectations.

	**AAE**		**MPE**		**IE**		**SAE**	
	***β* (SD)**		***β* (SD)**		***β* (SD)**		***β* (SD)**	
**Variables**	**1**	**2**	**1**	**2**	**1**	**2**	**1**	**2**
(Constant)	1.79^**^ (0.32)	1.78^**^ (0.32)	2.22^**^ (0.33)	2.19^**^ (0.33)	2.53^**^ (0.39)	2.49^**^ (0.39)	2.57^**^ (0.35)	2.54^**^ (0.35)
Sex	0.15^*^ (0.06)	0.16^*^ (0.06)	0.15^*^ (0.06)	0.15^*^ (0.06)	0.20^**^ (0.07)	0.21^**^ (0.07)	0.12 (0.07)	0.13 (0.07)
Type of household mobility	−0.07 (0.07)	−0.07 (0.07)	−0.18^*^ (0.07)	−0.18^*^ (0.07)	−0.13 (0.08)	−0.13 (0.08)	−0.15 (0.08)	−0.15^*^ (0.08)
Personnel structure	−0.06 (0.04)	−0.05 (0.04)	−0.05 (0.05)	−0.05 (0.05)	−0.00 (0.06)	0.00 (0.06)	−0.05 (0.07)	−0.04 (0.05)
Duration of residence	−0.11^*^ (0.05)	−0.10 (0.05)	−0.16^**^ (0.05)	−0.15^**^ (0.05)	−0.19^**^ (0.06)	−0.17^**^ (0.06)	−0.14^**^ (0.05)	−0.14^*^ (0.05)
SCS	−0.42^**^ (0.06)	−0.41^**^ (0.06)	−0.41^**^ (0.06)	−0.40^**^ (0.07)	−0.62^**^ (0.08)	−0.61^**^ (0.08)	−0.47^**^ (0.07)	−0.46^**^ (0.07)
ICMP	0.80^**^ (0.05)	0.77^**^ (0.05)	0.82^**^ (0.06)	0.82^**^ (0.06)	0.85^**^ (0.06)	0.85^**^ (0.06)	0.72^**^ (0.06)	0.72^**^ (0.06)
SCS*ICMP		0.15^**^ (0.07)		0.073 (0.07)		0.22^**^ (0.09)		0.07 (0.08)
**Δ*R*^2^**	0.76	0.77	0.76	0.77	0.77	0.77	0.71	0.71
**F**	224.40^**^	308.69^**^	222.06^**^	206.91^**^	223.67^**^	208.83^**^	197.11^**^	184.98^**^

Standard deviations in the brackets; 1 = a regression model including independent variables SCS and ICMP; 2 = a model adds the product term of SCS and ICMP on the basis of model 1.

* p < 0.05,

** p < 0.01.

#### The impact of migrant children's identification with the college matriculation policy on their educational expectations at the social level

##### Regression analysis of perceived social discrimination and the educational expectations of migrant children

As seen in Tables 3, 4, perceived social discrimination had a significant negative predictive effect on AAE (β = −0.59, *p* < 0.01), MPE (β = −0.59, *p* < 0.01), IE (β = −0.59, *p* < 0.01), and SAE (β = −0.59, *p* < 0.01). H4 is supported by the data.

##### Moderating effect of migrant children's identification with the college matriculation policy on the relationship between perceived social discrimination and educational expectations

As seen in Table 8, all dimensions passed the significance test. In particular, ICMP had the most significant positive predictive effect on AAE (β = 0.51, *p* < 0.01) and MPE (β = 0.51, *p* < 0.01). The product term of PSD and ICMP also had a high positive effect on AAE (β = 0.11, *p* < 0.01) and MPE (β = 0.09, *p* < 0.05). In addition, ICMP had a significant positive effect on IE (β = 0.47, *p* < 0.01) and SAE (β = 0.47, *p* < 0.01). And the product term of PSD and ICMP also had a significant positive effect on IE (β = 0.07, *p* < 0.05) and SAE (β = 0.03, *p* < 0.05). Therefore, H6d is supported by the data.

**Table 8:** Moderating effect of ICMP on the relationship between PSD and educational expectations.

	**AAE**		**MPE**		**IE**		**SAE**	
	***β* (SD)**		***β* (SD)**		***β* (SD)**		***β* (SD)**	
**Variables**	**1**	**2**	**1**	**2**	**1**	**2**	**1**	**2**
(Constant)	2.04^**^ (0.32)	2.02^**^ (0.32)	2.01^**^ (0.33)	2.04^**^ (0.34)	2.93^**^ (0.38)	2.91^**^ (0.39)	2.83^**^ (0.36)	2.42^**^ (0.36)
Sex	0.05 (0.06)	0.05 (0.06)	0.02 (0.06)	0.02 (0.06)	0.03 (0.07)	0.04 (0.07)	−0.01 (0.07)	−0.01 (0.07)
Type of household mobility	−0.01 (0.07)	0.01 (0.07)	−0.13^*^(0.07)	−0.12^*^(0.07)	−0.08 (0.08)	−0.05 (0.08)	−0.09 (0.08)	−0.08 (0.08)
Personnel structure	0.05 (0.04)	0.05 (0.04)	0.08 (0.05)	0.08 (0.05)	0.06 (0.05)	0.05 (0.05)	0.03 (0.05)	0.03 (0.05)
Duration of residence	−0.01 (0.05)	−0.08 (0.05)	−0.13^*^(0.05)	−0.13^*^(0.05)	−0.17^**^ (0.06)	−0.17^**^ (0.06)	−0.14^*^(0.05)	−0.14^*^(0.05)
PSD	−0.47^**^ (0.06)	−0.47^**^ (0.06)	−0.41^**^ (0.06)	−0.41^**^ (0.06)	−0.42^**^ (0.07)	−0.42^**^ (0.07)	−0.49^**^ (0.06)	−0.49^**^ (0.06)
ICMP	0.51^**^ (0.06)	0.48^**^ (0.06)	0.51^**^ (0.06)	0.51^**^ (0.06)	0.47^**^ (0.07)	0.48^**^ (0.07)	0.47^**^ (0.06)	0.48^**^ (0.06)
PSD*ICMP		0.11^**^ (0.05)		0.09^*^(0.05)		0.07^*^(0.06)		0.03^*^(0.06)
**Δ*R*^2^**	0.73	0.74	0.72	0.72	0.68	0.69	0.73	0.73
**F**	379.95^**^	372.76^**^	273.32^**^	266.36^**^	266.00^**^	260.47^**^	220.39^**^	218.19^**^

Standard deviations in the brackets; 1 = a regression model including independent variables PSD and ICMP; 2 = a model adds the product term of PSD and ICMP on the basis of model 1.

* p < 0.05,

** p < 0.01.

## Discussion

With the acceleration of urbanization and the emergence of a large number of migrant children, the education of migrant children has received increasing attention in society, and the educational expectations of migrant children have become a hot research topic. Relevant studies in China and abroad have analyzed the educational expectations of migrant children from different perspectives, such as individual academic performance (Rutchick et al., 2009), family social capital (Sewell and Shah, 1968; Yang, 2012), and class segregation in schools (Minello and Barban, 2012; Wu and Huang, 2017). Neither domestic nor foreign studies have clearly elucidated the impact of policy identification on the educational expectations of migrant children, and it is unknown whether migrant children's identification with the college matriculation policy has had a significant impact on their educational expectations. In this study, we selected children in different grades in the most representative areas as the research samples and explored how migrant children's identification with the college matriculation policy has affected their educational expectations from the perspective of ecosystem theory.

Previous studies have found that individual academic performance and family social capital have a significant positive impact on the educational expectations of migrant children (Rutchick et al., 2009; Kleinjans, 2010) and that class segregation in schools has a significant negative impact on the educational expectations of migrant children (Minello and Barban, 2012; Wu and Huang, 2017). While this study confirmed those conclusions, it also found that migrant children's identification with the policy indeed played a role. Specifically, for migrant children who were more able to accept and recognize local policies, their individual academic performance and family social capital had a greater positive impact on their educational expectations, and class segregation in schools had a smaller negative impact on their educational expectations. Conversely, for migrant children who were less able to accept and recognize local policies, the positive impact of their individual academic performance and family social capital on their educational expectations was smaller, and the negative impact of class segregation in schools on their educational expectations was greater.

In addition, empirical studies on the impact of perceived discrimination on migrant children rarely involve educational expectations but rather focus on psychological status (Berkel et al., 2010), cultural adaptation (Berry et al., 2006), or social integration (Zhang et al., 2016). However, the research conclusions from those studies effectively support the research conclusions of this study. In this study, perceived social discrimination had a significant negative impact on the educational expectations of migrant children. Additionally, this study found that under the moderating effect of policy identification, for migrant children who were more able to accept and recognize the local college entrance examination policy, perceived social discrimination had a less negative impact on their educational expectations.

This study further analyses the mechanism by which policy identification impacts the educational expectations of migrant children, a topic that has very important practical relevance for policy implementation and reform. Based on the results of the analysis, policy identification plays a role through the following three mechanisms. The first is a compensation mechanism through the “principle of justice”. To reflect the differential compensation of social public policies, some provinces have implemented relatively flexible high school and college entrance examination policies for migrant children, undoubtedly providing education opportunities, education fairness, and cultural capital for migrant children and promoting the establishment of positive education expectations. The second is a cultural mechanism driven by “promoting learning through examinations”. Selective examinations have powerful functions of “promoting learning through examinations” and can increase the enthusiasm for learning for thousands of young people. The third is an institutional mechanism through “urban–rural integration”. The relatively flexible entrance examination policy for migrant children not only facilitates the “urbanization” of migrant children but also provides the impetus for them to obtain higher human capital. In summary, disadvantaged lower-class children are more susceptible to positive psychological cues. If they receive effective support through mainstream social public policies, develop positive educational expectations and receive high-level education, social mobility is possible.

The research findings should be interpreted with caution. First, the paper focuses on the application of quantitative research methods, with less focus on the accumulation and analysis of qualitative interview data. The next step is to continue to collect and analyze relevant data. Second, due to the limitation of the experimental design, the consideration of possible influential variables in this paper are not comprehensive, such as the impact effects from students' grade and sex. We will include these factors in our next research. Third, the sample size needs to be expanded. In our next study, we want to use national survey platforms, such as the CEPS (China Education Panel Survey) and CFPS (China Family Panel Studies), to embed scales for the college entrance examination policy for migrant children to conduct research on a larger scale. Finally, the researchers established stable relationships with the existing survey subjects and hope to conduct a follow-up survey to further analyse whether the college matriculation policy for migrant children and their educational expectations ultimately affect the acquisition of education, the accumulation of cultural capital accumulation, and an increase in social status.

## Data availability statement

The data supporting the findings of this study are available from the corresponding author upon reasonable request.

## Ethics statement

This study was reviewed and approved by the Ethics Committee of Qingdao University. Written informed consent for participation was not required for this study in accordance with the national legislation and the institutional requirements.

## Author contributions

JX: data processing and manuscript writing. CL: research design, data collection, and modeling. Both authors contributed to the article and approved the submitted version.

## Funding

This study was supported by National Social Science Foundation (Education) Project (BIA190164) - Study on the Coupling Relationship Between the Extension of Local Universities to County Schools and Rural Revitalization.

## Conflict of interest

The authors declare that the research was conducted in the absence of any commercial or financial relationships that could be construed as a potential conflict of interest.

## Publisher's note

All claims expressed in this article are solely those of the authors and do not necessarily represent those of their affiliated organizations, or those of the publisher, the editors and the reviewers. Any product that may be evaluated in this article, or claim that may be made by its manufacturer, is not guaranteed or endorsed by the publisher.
